# Retrospective analysis of clinical characteristics and treatment of patients with immune checkpoint inhibitors-induced adrenal insufficiency

**DOI:** 10.3389/fonc.2025.1614223

**Published:** 2025-08-18

**Authors:** Xiao Huang, Lin Zhang, Chuantao Zhang, Huiyun Wang, Zhuhai Shao, Ning Liu, Shanai Song, Man Jiang, Helei Hou

**Affiliations:** ^1^ School of Medicine, Qingdao University, Qingdao, Shandong, China; ^2^ Department of Oncology, The Affiliated Hospital of Qingdao University, Qingdao, Shandong, China; ^3^ Department of Pharmacy, The Affiliated Hospital of Qingdao University, Qingdao University, Qingdao, China

**Keywords:** immune checkpoint inhibitors, immune-associated adverse events, adrenal insufficiency, isolated adrenal deficiency, hypophysitis

## Abstract

**Background:**

Immune checkpoint inhibitors (ICIs) are effective against solid tumors but can trigger immune-related adverse events (irAEs), including adrenal insufficiency (AI). Given its impact on treatment efficacy and patient quality of life, understanding the clinical characteristics and outcomes of ICI-induced AI (ICI-AI) is critical.

**Methods:**

We conducted a retrospective analysis of 46 patients diagnosed with ICI-AI at a single center (May 2019–July 2024) and reviewed clinical trials/real-world studies on ICI-AI.

**Results:**

The cohort included 22 cases of isolated adrenocorticotropic hormone deficiency (IAD), 23 of hypophysitis, and 1 of primary adrenal insufficiency (PAI). Median time to AI onset was 7.8 months (range: 1.5–27.4), with a median of 7 ICIs cycles (range: 1–21). Common symptoms were fatigue, anorexia, and nausea; comorbidities included hypothyroidism (41.3%) and hyponatremia (63%). No ACTH-deficient patients recovered during follow-up, but glucocorticoid replacement alleviated symptoms in most cases (45/46). The objective remission rate for underlying malignancies post-AI was 63%. Concurrent irAEs in other organs were rare (3 cases).

**Conclusion:**

The median time to AI onset and ICIs cycles administered are key indicators of AI development. Both IAD and hypophysitis are common secondary AI manifestations; glucocorticoid replacement enables safe ICIs continuation.

## Introduction

ICIs have revolutionized traditional treatment approaches and are now the standard of care for various cancers, including lung carcinoma (1), urothelial carcinoma (2), gastric carcinoma (3), renal cell carcinoma (4) and melanoma (5). The ICIs family comprises cytotoxic T-lymphocyte-associated protein 4 (CTLA-4), programmed cell death protein 1 (PD-1), and programmed death-ligand 1 (PD-L1) inhibitors, which enhance T-cell activation to exert anti-tumor effects, significantly improving treatment outcomes and survival rates in cancer patients (6). Despite their efficacy, ICIs are associated with an increased risk of irAEs (7), which differ from those associated with chemotherapy and targeted therapy (8). Specific irAEs can be severe or even life-threatening (8). Given their potential to compromise therapeutic outcomes and adversely affect patient quality of life, it is imperative to implement effective management strategies for irAEs to optimize patient safety and treatment efficacy.

The latency of irAEs varies significantly depending on the tumor type and the ICIs type. Common irAEs encompass a wide range of organ-specific toxicities, including pulmonary, cardiac, cutaneous, endocrine, hepatic, and gastrointestinal manifestations (9). AI is a relatively rare but clinically significant endocrine toxicity associated with ICIs. The incidence of AI induced by ICIs is about 0.6 - 1.7% in cancer patients and varies depending on the ICIs used (10–12). Compared to other irAEs, the risk of fatal AI with ICIs is lower (13). However, AI can develop into a life-threatening condition, particularly during adrenal crises, characterized by severe hypotension, electrolyte imbalances, and metabolic disturbances (14). Due to its low incidence and nonspecific symptoms, AI is often underdiagnosed or diagnosed late, exacerbating clinical conditions, especially in patients experiencing acute adrenal crises (15). Therefore, increased attention to AI diagnosis and management is essential.

This study aimed to evaluate the clinical characteristics of ICI-AI, including the median occurrence time and the number of treatment cycles associated with AI onset. Additionally, we searched for clinical trials and real-world studies of ICI-AI to explore its occurrence.

## Methods

### Patients

Patients were identified through our hospital database using keywords (“malignancy immunotherapy”, “adrenal insufficiency”, “hypophysitis”, “electrolyte disturbance”). We have identified this patient population who developed AI after ICIs therapy at the Affiliated Hospital of Qingdao University between May 2019 and July 2024. The ICIs included in this study comprised anti-PD-1 (nivolumab, pembrolizumab, sintilimab, camrelizumab, tislelizumab, toripalimab), anti-PD-L1 (durvalumab, bemarituzumab), and anti-PD-1/CTLA-4 dual inhibitors (cadonilimab). Clinical information included demographic data (age and gender), tumor type, PD-L1 expression status, AI onset time, medications used, and laboratory test results. This study was approved by The Institutional Review Board of the Affiliated Hospital of Qingdao University (Approval No. QYFYWZLL30096) and adhered to the principles of the Declaration of Helsinki. After the initial screening, data from 46 eligible patients were included in the analysis.

**Figure 1:**
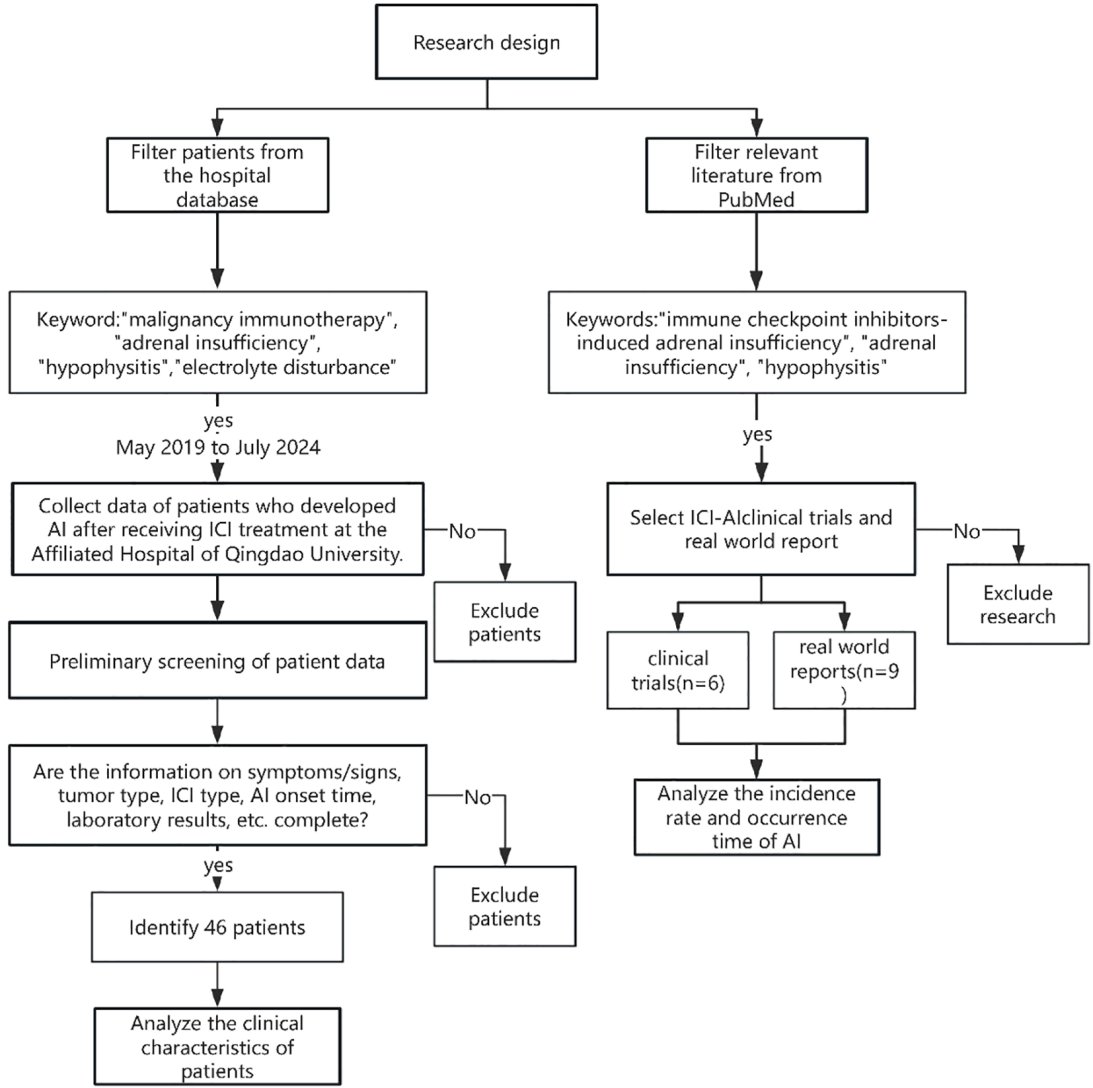
The flowchart of this research. AI, adrenal insufficiency; ICIs, Immune Checkpoint Inhibitors.

The major inclusion criteria for patients were as follows: (a) histologically confirmed solid malignancies treated with ≥1 cycle of ICIs; (b) new-onset adrenal insufficiency post-ICIs defined biochemically by morning basal cortisol <5 μg/dl and/or peak cortisol <18 μg/dl during 250 μg ACTH stimulation test; (c) diagnosis confirmed by endocrinology consultation; (d) complete clinical records including: tumor/ICIs details, serial cortisol/ACTH/electrolytes/thyroid function tests, hormone replacement regimens. The exclusion criteria for patients were as follows: (a) pre-existing pituitary/hypothalamic disorders (such as adenomas, metastases, sarcoidosis) or primary adrenal disease (such as autoimmune adrenalitis, bilateral adrenalectomy/radiotherapy, congenital hyperplasia); (b) AI by sepsis, adrenal hemorrhage; (c) incomplete clinical information including: unavailable ICIs treatment records, incomplete endocrine labs. **Figure 1** shows the flow chart of this study.

### Evaluation of AI/ACTH deficiency/thyroid-stimulating hormone deficiency

The diagnosis of AI was established based on early morning serum cortisol concentrations, which were below 5 μg/dl. ACTH deficiency was defined as low basal serum cortisol associated with low basal plasma ACTH concentrations (7.2 - 63.3 pg/mL). TSH deficiency was defined as low free thyroxine (fT4) (<12.8 pmol/L) and low or inappropriately normal TSH (0.75-5.6uIU/ml) (16). Adverse events were graded and evaluated according to the Common Terminology Criteria for Adverse Events (CTCAE) version 5.0.

### Diagnostic criteria for hypophysitis and IAD

To further classify ICI-AI, detailed subtyping was performed based on clinical presentation, imaging characteristics, and laboratory test results. The primary subtypes of SAI identified were hypophysitis and IAD.

Confirmation of hypophysitis requires either ≥1 pituitary hormone deficiency (including TSH or ACTH deficiency) with magnetic resonance imaging (MRI) abnormalities such as stalk thickening, suprasellar convexity, heterogeneous enhancement, and increased height of the gland, or ≥2 pituitary hormone deficiencies (including TSH or ACTH deficiency) accompanied by headache and other clinical symptoms (16).

IAD is characterized by a simultaneous decline in morning cortisol and ACTH levels, with other pituitary axes remaining unaffected (17). The diagnosis and management of these adverse events were conducted in consultation with endocrinologists or through involvement in multidisciplinary team (MDT) discussions.

### Screening for hypopituitarism

Usually, the baseline assessment was conducted within one week prior to the intervention initiation, followed by discontinuous screening accompanying tumor response evaluation. Additional assessments would be performed whenever clinical symptoms suggestive of hormone deficiency arose.

### Evaluation of tumor responses

According to the National Comprehensive Cancer Network (NCCN) guidelines or clinical trial studies, patients receive ICIs therapy every 2 to 3 weeks until disease progression or intolerable drug toxicity. Tumor responses were assessed by CT and/or MRI every 6 to 8 weeks or when signs of disease progression were observed, using the RECIST v1.1 criteria.

### Literature search and data extraction

We searched PubMed to identify clinical trials and real-world studies reporting ICI-AI, summarizing 6 clinical trials published ICI-AI cases and 9 real-world studies. The six included clinical trials (CheckMate 067, Keynote 091, Checkmate 238, Impower133, POSEIDON, ORIENT-15) reported AI incidence and severity, while the real-world studies provided selected data (first author, study type, tumor type, ICIs therapy, irAE type, latency) indicating SAI, including IAD and hypophysitis, as the primary manifestation.

### Statistical analysis

Categorical variables were presented as counts and percentages, while continuous variables were described as median and standard deviations. The data analysis was performed using IBM SPSS version 27.0 program.

## Results

The baseline characteristics of patients with ICI-AI are summarized in **Table 1**. The median time from ICIs initiation to AI onset was 7.8 months (range: 1.5 - 27.4 months), with a median of 7 ICIs cycles (range: 1 - 21). Notably, 65.2% of patients developed AI after 6 months of starting therapy. Most patients (45/46) were diagnosed with SAI, with only one patient diagnosed with PAI. Among the 45 patients with SAI, 22 were diagnosed with IAD, and 23 were diagnosed with hypophysitis.

**Table 1:** Baseline information of patients with ICI-AI.

Variables	Patients with AI (n = 46)
Age (range)	66 (52-82)
Sex (M/F)	38 (82.6%)/8 (17.4%)
Tumor type	
Lung cancer	23 (50%)
Gastric cancer	10 (21.7%)
Urothelial cancer	4 (8.7%)
Esophageal cancer	2 (4.3%)
Oropharynx cancer	2 (4.3%)
Others	5 (10.9%)
TPS<1% & CPS<1	5 (10.9%)
TPS ≥ 1% & CPS ≥ 1	23 (50%)
Unknown	18 (39.1%)
The grade of AI (CTCAE 5.0)	
Grade 1-2	7 (15.2%)
Grade 3-5	39 (84.8%)
Treatment of line	
First	30 (65.2%)
Second or ≥ third	4 (8.7%)
Adjuvant therapy	9 (19.6%)
Neoadjuvant therapy	3 (6.5%)
Monotherapy	6 (13%)
Combination therapy	40 (87%)
Other irAEs	
Hyponatremia	29 (63%)
Hypothyroidism	19 (41.3%)
Hypokalemia	7 (15.2%)
Hyperkalemia	2 (4.3%)
Eosinophilia	2 (4.3%)
Immune related pneumonia	1 (2.2%)
Immune related dermatitis	1 (2.2%)
Immune related enteritis	1 (2.2%)
Pituitary MRI	
Normal	12 (26.1%)
Abnormal	9 (19.6%)
Unknown	25 (53.3%)

AI, adrenal insufficiency; F, Female; ICI-AI, Immune checkpoint Inhibitors-induced adrenal insufficiency; irAEs, immune-related adverse events; M, Male; MRI, magnetic resonance imaging; TPS, Tumor Proportion Score; CPS, Combined Positive Score; CTCAE, Common Terminology Criteria for Adverse Events.

The most common type of cancer was lung cancer (n=23), followed by gastric cancer (n=10), urothelial cancer (n=4), esophageal cancer (n=2), oropharyngeal cancer (n=2), and other malignancies (small intestine cancer, gallbladder cancer, hepatocellular carcinoma, and oral cancer). Among the 28 patients with known PD-L1 expression status, 23 showed PD-L1 expression ≥1%. Regarding AI severity, 15.2% of patients had grade 2 (G2) AI, 82.6% had grade 3 - 4 (G3 - 4) AI, and 2.2% had grade 5 (G5) AI. Patients received ICIs primarily in the first line (n=30), second line/higher line settings (n=4), neoadjuvant (n=3), and adjuvant treatments (n=9). Forty patients received ICIs in combination with other treatments, while only six patients received ICIs monotherapy.

As shown in **Supplementary Table S1**, forty patients received PD-1 inhibitors (11 sintilimab, 11 pembrolizumab, 9 camrelizumab, 3 tislelizumab, 3 nivolumab, 2 toripalimab, 1 serplulimab). Two patients received a PD-L1 inhibitor (durvalumab, bemarituzumab), one received a PD-1/CTLA-4 bispecific inhibitor (cadonilimab), and three patients received sequential treatments combining two types of ICIs. The clinical symptoms and signs at diagnosis were nonspecific and consistent across patients (**Supplementary Table S2**). The most common complaint was fatigue (73.9%), followed by anorexia (65.2%), nausea (45.7%), and vomiting (23.9%). Twenty-five patients stopped ICIs therapy due to AI, and seven patients had already discontinued it due to completion of the treatment course or other reasons. Among the former, six patients were unable to resume immunotherapy due to disease progression, and 19 patients stopped due to drug toxicity. Except for patient 13, who had already received glucocorticoid replacement due to immune-related pneumonia, other patients had not received glucocorticoid replacement therapy before. All patients received glucocorticoid replacement, consistent with the recommended dosing for endocrine irAEs in China’s expert consensus. Glucocorticoid replacement was effective for all patients except one who died from severe hyponatremia-induced cerebral edema. All patients who developed AI received lifelong glucocorticoid replacements. After glucocorticoid replacement, 14 patients resumed ICIs therapy with glucocorticoid replacement as the foundation.

Hyponatremia was the most common electrolyte disturbance, and hypothyroidism was the most common co-occurring irAE. Additionally, three of the 46 patients (6.5%) experienced other co-occurring irAEs, including skin toxicity (n=1), lung toxicity (n=1), and gastrointestinal toxicity (n=1). Patient 13 developed ICI-pneumonia two months before the onset of AI. Patient 40 developed ICIs-rash one month after the onset of AI, with symptoms improving after methylprednisolone treatment. Patient 27 developed ICIs-enteritis six months after the onset of AI, with symptoms improving after glucocorticoid replacement. Nine patients had abnormalities of the pituitary gland: two patients showed enlargement of the pituitary gland, six patients had signal enhancement of the gland, and one patient presented a partial empty sella turcica.

The comparative analysis of hypophysitis and IAD induced by ICIs is summarized in **Table 2**. Among the 23 patients diagnosed with hypophysitis, the median onset time was 7.7 months, compared to 6.7 months in the 22 patients diagnosed with IAD. Fatigue was the most common symptom in both groups (87% in hypophysitis vs. 63.7% in IAD), followed by anorexia (69.6% vs. 59.1%) and nausea (34.8% vs. 50%). Notably, headache (17.4%) and visual disturbances (13%) were observed exclusively in hypophysitis, likely due to pituitary inflammation and mass effect. All patients in both groups exhibited deficiencies in the ACTH-cortisol axis. However, hypophysitis patients frequently presented with additional hormonal deficiencies, including reduced TSH (26.1%) and fT4 (78.3%), whereas these axes remained normal in IAD. Among patients with available MRI data (excluding unknown cases), pituitary enlargement (36.4%) and abnormal signal intensity (45.5%) were observed in hypophysitis, whereas all IAD patients had normal pituitary imaging.

**Table 2:** Comparative Features of Hypophysitis and IAD.

Feature	Hypophysitis (n=23)	IAD(n=22)
Definition	Pituitary inflammation causing multiple hormone deficiencies	Isolated glucocorticoid deficiency (ACTH-cortisol axis only)
Onset	median: 7.7 months/7 cycles	median: 6.7 months/8.5 cycles
Key Symptoms	Fatigue (87%) Anorexia (69.6%) Nausea (34.8%) Headache (17.4%) visual disturbances (13%)	Fatigue (63.7%) Anorexia (59.1%) Nausea (50%) Headache (0%) visual disturbances (0%)
Hormonal Deficiencies		
ACTH/Cortisol	↓↓ ACTH, ↓↓ Cortisol (100%)	↓ ACTH, ↓ Cortisol (100%)
Other Axes Involved	TSH↓ (26.1%), fT4↓ (78.3%)	Normal (TSH, fT4)
MRI Findings	Pituitary enlargement (36.4%) (Excluding unknown) Pituitary abnormal signal (45.5%) (Excluding unknown)	Normal pituitary (100%) (Excluding unknown)
Critical Management	Glucocorticoids ± hormone replacement	Glucocorticoid replacement only
Adrenal Crisis	None	None

ACTH, adrenocorticotropic hormone; fT4, free thyroxine; IAD, isolated adrenocorticotropic hormone deficiency; MRI, magnetic resonance imaging; TSH, thyroid-stimulating hormone.

Using PubMed, we searched for clinical trials reporting ICI-AI and identified 6 published trials involving ICI-AI; these are summarized in **Table 3**. All the 6 clinical trials reported the numbers and the severity of AI. Due to the main purpose of all trials being to evaluate efficacy, there was no systematic analysis of the clinical characteristics and occurrence time of drug toxicity. No trial reported the deaths attributed to ICI-AI. ICIs was administered as monotherapy (3 trials) (CheckMate 067, Keynote 091, Checkmate 238), in combination with chemotherapy (3trials) (Impower133, POSEIDON, ORIENT-15). The incidence of AI among patients across all trials was less than 2%. We did not find large-scale clinical trial reports on the occurrence of AI related to camrelizumab, trastuzumab, and triprolizumab.

**Table 3:** Comparison of AI occurrence between different ICIs in clinical trials.

Study	Tumor type	ICIs	Stage	irAE type	Patients	Incidence rate	Grade 1–2	Grade 3–4
CheckMate 067 (47)	Melanoma	Nivolumab	IV	AI	313	4(1.3%)	2(0.6%)	2(0.6%)
CheckMate 067 (47)	Melanoma	Ipilimumab	IV	AI	311	4(1.3%)	3 (1%)	1 (<1%)
Impower133 (48)	SCLC	Atezolizumab+EC	Extensive	None	2291	0	0	0
Keynote-091 (11)	NSCLC	Pembrolizumab	IB-IIIA	AI	580	10(1.7%)	6 (1%)	4 (0.7%)
POSEIDON (12)	NSCLC	Duvalimumab+chemotherapy	IV	AI	334	4(1.2%)	3(0.9%)	1(0.3%)
Checkmate 238 (49)	Melanoma	Ipilimumab	IIIC-IV	Hypophysitis	453	1 (<1%)	1 (<1%)	0
ORIENT-15 (10)	OSCC	Sintilimab+chemotherapy	III-IV (locally advanced)	Hypophysitis	328	2 (0.6%)	1 (0.3%)	1 (0.3%)

AI, adrenal insufficiency; EC, Etoposide and Carboplatin; ICIs, Immune Checkpoint Inhibitors; irAE, immune-related adverse events; NSCLC, non-small cell lung cancer; OSCC, oesophageal squamous cell carcinoma; SCLC, small cell lung cancer.

We searched PubMed for real-world studies reporting ICI-AI and identified 9 relevant published studies, which are summarized in **Table 4**. The following information was selected: first author, study type, tumor type, ICIs therapy, irAE type, and latency of ICI-AI. SAI is the main manifestation of patients receiving immunotherapy, including IAD and hypophysitis. The median time from the initiation of ICIs to AI onset varies from several weeks to several months. The frequency of AI occurrence in real-world research was higher than in large-scale clinical studies.

**Table 4:** Comparison of AI occurrence between different ICIs in real world reports.

Author	Study type	Tumor type	Patients	ICIs therapy	irAE type	Incidence rate	Latency
Eva Kassi et al,2019 (20).	prospective	Melanoma	339	CTLA4	Hypophysitis	5(4.2%)	22 weeks (4-156)
				PD1/PDL1		7(5.5%)	
				CTLA4+ PD1/PDL1		9(9.8%)	
Qingqing Cai et al,2024 (50).	retrospective	Liver cancer	419	PD1	AI	47(11.2%)	190 days (21-570)
Ruth Percik M.D et al,2020 (21)	retrospective	Multiple types of cancer	1615	CTLA4+ PD1/PDL1	IAD	14 (0.87%)	5.8 months
					Hypophysitis	6(0.37%)	
Qingqing Cai et al,2023 (51).	retrospective	Multiple types of cancer	1241	CTLA4+ PD1/PDL1	PAI	7(0.6%)	200.5 days (35-280)
					SAI	94(7.6%)	178 days (16-562)
Jing Xiang et al,2023 (37).	retrospective	Multiple types of cancer	1014	PD1/PDL1	AI	20 (2%)	5.2 months (3.0-7.5)
Alexander T. Faje et al,2023 (45).	cohort study	Melanoma	154	CTLA4	Hypophysitis	17 (11%)	8.4 months (6.9 –10.3)
Stephanie van der Leij et al,2024 (52).	retrospective	Multiple types of cancer	67	PD1/PDL1	Hypophysitis	NA	22 weeks
				CTLA4			11 weeks
				CTLA-4/PD-1			14 weeks
Tomoko Kobayashi et al,2021 (53).	prospective	Multiple types of cancer	62	CTLA4 PD1/PDL1	Hypophysitis IAD	5(8.1%) 17(27.4%)	NA NA
Jake Johnson et al,2023 (54).	retrospective	Multiple types of cancer	839	PD1/PDL1	Hypophysitis	8(1%)	7 months
				CTLA4		1(0.1%)	
				CTLA-4/PD-1		7(0.8%)	

AI, adrenal insufficiency; CTLA-4, Cytotoxic T-lymphocyte-associated Protein 4; ICIs, Immune Checkpoint Inhibitors; irAE, immune-related adverse events; IAD, isolated adrenocorticotropic hormone deficiency; PAI, primary adrenal insufficiency; SAI, secondary adrenal insufficiency; PD-1, Programmed Cell Death Protein 1; PD-L1, Programmed Death Ligand 1.

## Discussion

AI associated with ICIs primarily includes PAI, IAD, and hypophysitis. Previous studies report that the incidence of AI induced by CTLA-4 inhibitors is approximately 4% (18–20). The incidence of AI in patients receiving PD-1/PD-L1 Inhibitors was about 0.4% (21, 22). Studies have shown the risk of developing AI in patients receiving combination therapy was higher than patients with monotherapy (23). A prospective study showed that CTLA-4 inhibitors caused IAD and hypophysitis, while PD-1 or PD-L1 inhibitors only caused IAD (24). However, there was no difference in our study in the frequency of hypophysitis and IAD induced by PD-1 inhibitors.

The onset of ICI-AI typically occurred later, with a median latency of 7.8 months, compared to a 6-week latency for dermatologic toxicities, a 14.7-week latency for pneumonitis toxicity, and an 8-week latency for myocardial toxicity (25–27). This delayed onset can lead to challenges in timely clinical diagnosis. However, some patients developed AI in the early stages of ICIs therapy. In this study, the shortest latency was 1.5 months, and the longest latency was 27.4 months. ICI-AI could occur at any time after ICIs therapy. Therefore, it is necessary to screen for important laboratory tests and imaging examination, such as electrolytes, morning cortisol, ACTH and MRI, even after cessation of ICIs treatment. Significantly, the dynamic progression of ICI-AI on MRI typically manifests as glandular swelling in the acute phase, which may later progress to atrophy in the chronic phase. Consequently, even in cases where pituitary MRI findings appear normal, clinicians should remain vigilant for the potential development of AI.

The clinical manifestations of ICI-AI were often nonspecific and common (30).

Chemotherapy can also induce adverse effects including nausea and vomiting, fatigue, dehydration, electrolyte imbalances, infection, pain, and adverse effects of opioids. This nonspecific symptomatology can readily mask underlying AI. Consequently, maintaining a high index of clinical suspicion is paramount. AI must be actively considered in the differential diagnosis for cancer patients presenting with persistent or refractory symptoms such as fatigue, dizziness, gastrointestinal disturbances, unexplained hypotension, or hyponatremia. This is particularly crucial for patients with risk factors, including known bilateral adrenal metastasis, a history of surgery near the hypothalamic-pituitary axis, or immunotherapy. Biochemical confirmation via dynamic function testing (such as ACTH stimulation test) remains essential, as reliance on symptoms alone is inadequate.

Common hyponatremia and hypothyroidism in AI were significantly correlated with AI (31–34). It is important to note that exogenous thyroid hormone can expedite cortisol clearance, increasing the likelihood of an adrenal crisis. Consequently, patients with both hypothyroidism and AI should be treated with glucocorticoid replacement prior to initiation of thyroid hormone replacement therapy. Studies showed that new-onset eosinophilia was also closely related to AI (35–37). In our study, only two patients with AI had eosinophilia, which may be related to the small sample size. Hypothyroidism, hyponatremia, and newly developed eosinophilia may be early predictive markers that contribute to the early identification of AI. Further work is needed to explore the early predictions of AI.

Patients diagnosed with AI require lifelong glucocorticoid replacement therapy. The commonly used medications in clinical practice are hydrocortisone (15 - 25 mg) or prednisone (20 - 35 mg), administered 2 - 3 times daily (38). Related studies have shown that high-dose glucocorticoid replacement did not restore pituitary function (38–40) and can inhibit TSH secretion (41). In addition, long-term use of high-dose glucocorticoids can increase the risk of respiratory and gastrointestinal infections (42). Therefore, high doses of glucocorticoids are only recommended for patients with life-threatening adrenal crisis and impaired consciousness (43). For most patients, long-term physiological hormone replacement therapy is sufficient. Notably, in this study, AI did not recur upon restarting ICIs therapy. Regarding immunotherapy recovery after the occurrence of AI, this study showed that ICIs therapy could be restarted with glucocorticoid replacement. This differs from other serious irAEs, such as ICIs-pneumonia, where ICIs therapy is typically terminated (28), and ICIs-myocarditis, where current guidelines recommend permanent discontinuation of ICIs therapy (29). Clinicians need to adjust the dosage according to the patient’s symptoms and condition. Therefore, regular screening is crucial.

Previous research indicated a significant correlation between ICI-AI and ICIs therapy efficacy in cancer patients (44). Other studies also showed a positive association between IAD/hypophysitis and overall survival (45, 46). Therefore, the development of AI during ICIs therapy may serve as a positive predictive indicator of treatment response.

The comparative data highlight distinct clinical profiles between hypophysitis and IAD, emphasizing the importance of imaging and hormonal profiling for accurate diagnosis. The presence of symptoms like headache and visual disturbances in hypophysitis, alongside multi-axis hormonal deficiencies, underscores the broader inflammatory impact on the pituitary gland. In contrast, IAD’s restriction to the ACTH-cortisol axis and normal pituitary imaging supports its classification as a more targeted dysfunction. The absence of adrenal crisis in both groups is notable, possibly reflecting timely diagnosis and intervention in our cohort. However, the higher prevalence of pituitary abnormalities in hypophysitis (45.5%) reinforces the need for routine MRI evaluation in symptomatic patients to guide management. The therapeutic divergence — glucocorticoids alone for IAD versus broader hormone replacement in hypophysitis — emphasizes the importance of accurate differentiation between these conditions to optimize patient treatment.

This study has several limitations, including its retrospective design, single-center nature, and small sample size. These findings introduce new questions, such as whether the timing of onset correlates with specific ICIs or preexisting risk factors. Future research should explore longitudinal outcomes and biomarkers to refine predictive and diagnostic strategies for ICIs-induced endocrine complications.

## Conclusions

In summary, the median time to AI onset and the number of ICIs therapy cycles are critical factors in understanding the temporal patterns of AI development. Both IAD and hypophysitis are common manifestations of SAI induced by ICIs. Patients diagnosed with AI can safely resume ICIs therapy with appropriate glucocorticoid replacement. Regular monitoring of cortisol levels following the administration of immune checkpoint inhibitors is essential for early detection and management of AI.

## Data Availability

The raw data supporting the conclusions of this article will be made available by the authors, without undue reservation.
