# Supplementary material for: Retrospective analysis of clinical characteristics and treatment of patients with immune checkpoint inhibitors-induced adrenal insufficiency
Source: Front Oncol. 2025 Aug 18;15:1614223. doi: 10.3389/fonc.2025.1614223 (PMC12409743; doi:10.3389/fonc.2025.1614223)
Supplement: Supplementary file 1 [file DataSheet1.pdf]

Table S1 Detailed clinical profiles of anti-tumor drugs and the time of AI onset in 46 patients.

No.	Tumor type	Stage	Immune checkpoint inhibitors		Other drugs	AI onset	
			name	Dose(mg)/cycle		cycle	time
1	LUSC	IV	Toripalimab	240 mg/3weeks	AN	14	14.5
2	LUAD	IV	Camrelizumab	200 mg/3weeks	Pem+Bev	11	12.3
3	SCLC	IV	Duvalimumab	1500 mg/3weeks	EC	4	4.6
4	STAD	IV	Sintilimab	200 mg/3weeks	Paclitaxel-albumin+cap	4	4.6
5	LSCA	IV	Pembrolizumab	200 mg/3weeks	AN+ anlotinib	7	14.9
6	LUSC	IV	Pembrolizumab	200 mg/3weeks	TC	6	4.7
7	Small intestine	IV	Sintilimab→Durvalimumab	200 mg/3weeks→1000 mg/3weeks	oxaliplatin+Raltitrexed	21	19.8
8	LUSC	IIIA	Pembrolizumab	200 mg/3weeks	TP	16	13.5
9	SCLC	IV	Serplulimab	300 mg/2weeks	EC	5	6.9
10	LUAD	IV	Camrelizumab	200 mg/3weeks	TP	5	5.5
11	STAD	IV	Pembrolizumab	200 mg/3weeks	FOLFOX	13	14.6
12	STAD	IV	Nivolumab	360 mg/3weeks	SOX	5	4.3
13	LUAD	IV	Pembrolizumab→Torip	200 mg/3weeks→2	anlotinib	10	12.0

			alimab	40mg/3weeks			
14	LUAD	IV	Camrelizumab	200 mg/3weeks	PC+Bev	13	24.6
15	GACA	IIA	Camrelizumab	200 mg/3weeks	Tegafur	2	6.0
16	ORSC	II	Camrelizumab	200 mg/3weeks	Chemotherapeutics	10	7.8
17	LUSC	IIIB	Pembrolizumab	200 mg/3weeks	GP	19	27.2
18	LUAD	IIIA	Pembrolizumab	200 mg/3weeks	/	4	3.3
19	LUSC	IV	Pembrolizumab	200 mg/3weeks	TC	10	8.8
20	LUAD	IIIB	Pembrolizumab	200 mg/3weeks	PC	18	21.3
21	SKSC	IV	Cadonilimab	625 mg/3weeks	/	9	14.0
22	ESCA	IV	Sintilimab	200 mg/3weeks	TP	3	27.4
23	LIHC	IV	Camrelizumab	200 mg/3weeks	Sorafenib	9	10.5
24	ORSC	III	Pembrolizumab	200 mg/3weeks	Nimotuzumab	9	6.2
25	LUSC	IV	Tislelizumab	200 mg/3weeks	TC	16	18.8
26	LUSC	IV	Sintilimab	200 mg/3weeks	ANRHE	7	6.1
27	STAD	IB	Pembrolizumab	200 mg/3weeks	/	8	7.7

28	LUAD	IV	Sintilimab	200 mg/3weeks	Pem+cap	19	15.1
29	STAD	IIIC	Nivolumab	360 mg/3weeks	XELOX	9	8.1
30	UC	III	Tislelizumab→ Sintilimab	200 mg/3weeks	Epirubicin	4	2.1
31	STAD	IV	Sintilimab	200 mg/3weeks	SOX	17	17.0
32	Melanoma	IV	Pembrolizumab	200 mg/3weeks	TC	9	10.1
33	GSRCC	IV	Sintilimab	200 mg/3weeks	TP	7	6.8
34	UC	I	Sintilimab	200 mg/3weeks	/	6	6.5
35	UC	II	Toripalimab	240 mg/3weeks	GP	2	1.7
36	LSCA	IIIA	Sintilimab	200 mg/3weeks	/	5	6.9
37	LUSC	IV	Tislelizumab	200 mg/3weeks	TC	4	3.7
38	ESCA	IV	Sintilimab	200 mg/3weeks	AN	1	1.5
39	BLCA	II	Tislelizumab	200 mg/3weeks	/	6	9.4
40	STAD	III	Nivolumab	300 mg/3weeks	XELOX	2	2.7
41	LUAD	IV	Camrelizumab	200 mg/3weeks	PC	14	18.5
42	LUSC	IV	Camrelizumab	200 mg/3weeks	AN+RHE	9	7.5
43	LUSC	IIIB	Camrelizumab	200 mg/3weeks	TC	8	3.9
44	SCLC	IV	Bemarituzu	1200mg/3week	EC	6	5.6

			mab	s			
45	STAD	IV	Sintilimab	200 mg/3weeks	FOLFOX	12	10.5
46	STAD	IV	Sintilimab	200 mg/3weeks	TC	5	4.4

AI:adrenal insufficiency; AN:albumin paclitaxel and nedaplatin; Bev: Bevacizumab; BLCA:bladder urothelial carcinoma; cap:capecitabine; EC:etoposide and carboplatin; ESCA:esophageal carcinoma; FOLFOX:oxaliplatin and calcium folinate and fluorouracil; GACA:gallbladder carcinoma; GP: gemcitabine and cisplatin; GSRCC:gastric signet ring cell cancer; LIHC:liver hepatocellular carcinoma; LSCA:lung sarcomatoid carcinoma; LUAD:lung adenocarcinoma; LUSC:lung squamous cell carcinoma; ORSC:oropharyngeal squamous cell carcinoma; PC:pemetrexed and cisplatin; RHE:recombinant human endostatin; SOX:oxaliplatin and tegafur; SCLC:small cell lung cancer; STAD:stomach adenocarcinoma; TC:albumin paclitaxel and carboplatin; TP:albumin paclitaxel and cisplatin; SKSC:skin squamous cell carcinoma; UC:urothelial carcinoma; Pem:Pemetrexed; XELOX:oxaliplatin and capecitabine.

Table S2 Clinical characteristics of 46 patients with ICI-AI.

No.	Symptoms	CORT (mmol/l)	ACTH (pg/ml)	Laboratory abnormalities	Hormone therapy (mg)	Re-administration	Grade
1	Fatigue,nausea,chest tightness	8.53	<1.5	Hyponatremia, hypothyroidism	Prednisolone 7.5	-	4

				,hypokalemia			
2	Fatigue,anorexia,nausea, weight loss	6.47	<1.5	Hyponatremia	Hydrocortisone 40	-	4
3	Giddiness,eft eyeball fixation, blepharoptosis	14	<1.5	hypothyroidism	Prednisolone 5	+	3
4	Fatigue, anorexia	14.2	3.03	Hyponatremia, hypothyroidism	Hydrocortisone 30	-	3
5	Nausea,vomiting,giddiness	28	>2000	Hyponatremia, hyperkalemia	Methylprednisolone 40	-	5
6	Fatigue,anorexia	9.57	2.95	hypokalemia	Prednisolone 7.5	+	2
7	Fatigue,anorexia	2.88	<1.5	Hyponatremia, hypothyroidism	Prednisolone 7.5	+	3
8	Fatigue,anorexia	7.59	1.96	Hyponatremia, hypothyroidism	Hydrocortisone 30	-	2
9	Fatigue,anorexia, nausea,vomiting	6.48	5.72	Hyponatremia, hypothyroidism	Hydrocortisone 20	-	3
10	Fatigue,nausea,vomiting	<1.5	<1.5	/	Prednisolone 10	-	3
11	Fatigue,anorexia	67.3	18.5	Hyponatremia, hypothyroidism	Hydrocortisone 20	-	3
12	Fever, anorexia	27.4	<1.5	Hyponatremia	Prednisolone 7.5	-	3
13	Fatigue,nausea,giddiness	<1.5	12.5	Hyponatremia, hyperkalemia	Methylprednisolone 16	-	3
14	Fatigue,anorexia	2.38	<1.5	Hypothyroidism	Hydrocortisone 20	+	3
15	Anorexia,nausea, vomiting	1.63	<1.5	/	Prednisolone 2.5	-	3

16	Chest tightness, holding breath	31	<1.5	Hyponatremia, hypokalemia	Methylprednisolone 12	-	3
17	Fatigue	12.18	15.16	/	Methylprednisolone 8	+	3
18	Fatigue,anorexia,nausea	7.32	5.04	eosinophilia	Prednisolone 5	+	3
19	Fatigue,nausea,vomiting,giddiness,diarrhea	9.12	14.4	Hyponatremia	Hydrocortisone 30	+	3
20	Anorexia	2.94	1.99	/	Prednisolone 7.5	+	3
21	Anorexia,nausea, vomiting,giddiness, diarrhea	17.7	15.3	Hyponatremia	Hydrocortisone 30	-	3
22	Fatigue,headache	5.04	<1.5	Hypokalemia,hypothyroidism	Prednisolone 5	-	3
23	Fatigue,anorexia, headache	5.95	2.21	Hypokalemia,hypothyroidism	Hydrocortisone 30	-	2
24	Fatigue	16.7	17.5	Hyponatremia	Prednisolone 7.5	+	2
25	Fatigue,anorexia	37.5	12.2	Hyponatremia	Hydrocortisone 30	+	3
26	Anorexia,nausea,limb numbness	5.12	<1	Hyponatremia, hypothyroidism	Prednisolone 7.5	-	3
27	Fatigue,anorexia,nausea, vomiting	5.34	2.19	Hyponatremia, hypothyroidism	Hydrocortisone 30	+	3
28	Fatigue,anorexia,weight loss,diarrhea	15.21	<1	Hyponatremia, hypothyroidism	Prednisolone 7.5	+	3
29	Fatigue,anorexia,nausea, weight loss	25.02	2.93	Hyponatremia	Prednisolone 7.5	-	3
30	Fatigue, anorexia,nausea	16.8	<1.5	Hyponatremia	Hydrocortisone30	-	3

31	Fatigue,nausea,vomiting,giddiness,headache	5.45	<1.5	Hyponatremia, hypothyroidism	Prednisolone 7.5	-	2
32	Giddiness,nausea,listlessness	7.91	4.36	/	Hydrocortisone 30	-	2
33	Fatigue, anorexia	6.51	<1.5	/	Methylprednisolone 8	-	3
34	Fatigue, anorexia,nausea, vomiting,giddiness, chest tightness, holding breath	47.4	4.34	Hyponatremia, hypokalemia	Hydrocortisone 30	-	3
35	Nausea,vomiting	8.25	7.82	/	Hydrocortisone 80	-	3
36	/	11.6	20.4	/	Prednisolone 5	-	2
37	Fatigue	5.8	<1.5	Hyponatremia, hypothyroidism	Hydrocortisone 30	-	3
38	Fatigue, anorexia	68.2	<1.5	/	Methylprednisolone 80	-	3
39	Unconsciousness	0.07	1.84	/	Prednisolone 15	-	4
40	Fatigue,anorexia,nausea, vomiting	10.1	<1.5	Hyponatremia, hypothyroidism	Hydrocortisone30	-	4
41	Fatigue,anorexia	2.19	2.6	Hyponatremia, hypothyroidism	Prednisolone 5	+	3
42	Fatigue,anorexia,weight loss	2.76	1.54	Hyponatremia, hypothyroidism	Methylprednisolone 8	-	3
43	Fatigue,anorexia,nausea, giddiness	3.49	16	Hypokalemia,eosinophilia	Prednisolone 5	+	3
44	Fatigue,anorexia	132	＜1.5	Hyponatremia, hypothyroidism	Hydrocortisone 30	-	3

45	Fatigue,anorexia,nausea	13.4	＜1.5	Hyponatremia	Prednisolone 5	-	3
46	Fatigue,anorexia	4.55	＜1.5	Hyponatremia	Prednisolone 5	-	3

ICI-AI:Immune checkpoint Inhibitors-induced adrenal insufficiency; ACTH:adrenocorticotropic hormone.
